# Chemical Mechanical Planarization and Old Italian Violins

**DOI:** 10.3390/mi9010037

**Published:** 2018-01-18

**Authors:** Ara Philipossian, Yasa Sampurno, Lauren Peckler

**Affiliations:** 1 Araca, Inc., Tucson, AZ 85718, USA; yasayap@email.arizona.edu (Y.S.); 2 Department of Chemical and Environmental Engineering, University of Arizona, Tucson, AZ 85721, USA; razzie84@email.arizona.edu (L.P.)

**Keywords:** chemical mechanical planarization (CMP), spectral analysis of sound, spectral analysis of shear forces, force cluster plots, violin, guarneri, directivity, mobility

## Abstract

Previous studies have shown that spectral analysis based on force data can elucidate fundamental physical phenomena during chemical mechanical planarization (CMP). While it has not been literally described elsewhere, such analysis was partly motivated by modern violinmakers and physicists studying Old Italian violins, who were trying to discover spectral relations to sound quality. In this paper, we draw parallels between violins and CMP as far as functionality and spectral characteristics are concerned. Inspired by the *de facto* standard of violin testing via hammer strikes on the base edge of a violin’s bridge, we introduce for the first time, a mobility plot for the polisher by striking the wafer carrier head of a CMP polisher with a hammer. Results show three independent peaks that can indeed be attributed to the polisher’s natural resonance. Extending our study to an actual CMP process, similar to hammered and bowed violin tests, at lower frequencies the hammered and polished mobility peaks are somewhat aligned. At higher frequencies, peak alignment becomes less obvious and the peaks become more isolated and defined in the case of the polished wafer spectrum. Lastly, we introduce another parameter from violin testing known as directivity, Δ, which in our case, we define as the ratio of shear force variance to normal force variance acquired during CMP. Results shows that under identical polishing conditions, Δ increases with the polishing removal rate.

## 1. Introduction and Motivation

For the past 450 years, the one thousand or so surviving instruments made by the Cremonese master luthiers such as Andrea Amati (1505–1577), Antonio Stradivari (1644–1737) and Bartolomeo Guiseppe Guarneri del Gesù (1698–1749) have entertained listeners worldwide. At the same time, they have also continued to amaze and inspire not only professional musicians, but also modern luthiers, physicists, biologists, chemists, archeologists, optical scientists, musicologists, mechanical engineers and material scientists, just to name a few. The German physicist, Hermann Backhaus, was one of the first to study vibration patterns in Old Italian violins [1,2,3]. His work was continued on by Saunders [4], Cremer [5], Schelleng [6], Moral [7] and Hutchins [3], culminating in further breakthroughs in recent years thanks to works by Dunnwald [8], Jansson [9,10,11], Harris [12], Buen [13,14], Morset [15], Bissinger [16,17], and Curtin and Rossing [18]. Zwicker [19] and Stepanek [20] were two of the first musicologists to correlate spectral relations to the psychoacoustic aspects of sound quality, while Guettler published groundbreaking work on the properties of rosin and how they may affect stick-slip events during playing [21,22].

In this paper, we focus on the subject of chemical mechanical planarization (CMP) and how the mechanical and kinematic aspects of the process may analytically and functionally relate to the violin. In today’s technology, all electronics incorporate integrated circuits (IC) consisting of tens of billions of connected transistors fabricated on one square centimeter of a single-crystalline silicon wafer. To achieve such a complex network of interconnects, there must exist multiple layers of conductors and insulators (presently, there are more than 20 layers of each) above the transistors [23]. ICs are fabricated “bottom-up” through sequential layering processes. Fabricating each layer causes severe topography, which in order to achieve the needed depth for focus requirements, has to be made more or less optically flat prior to forming any subsequent layers above it. To achieve such planarized wafer surfaces, CMP was invented and used in manufacturing by International Business Machines (IBM) in the mid-1980s [24]. Since then, CMP has been widely used in IC manufacturing to achieve both local and global planarization and has become one of the main elements in enabling Moore’s Law [25].

During CMP, a wafer is pressed against a rotating polyurethane pad with slurry being delivered on top of the pad surface (typically near its center). In many cases, a softer sub-pad is installed in between the pad and the platen in order to improve global planarity across the entire 300 mm diameter wafer. The slurry contains nano-sized abrasive silica or ceria particles and a plethora of chemicals depending on the specific polishing process. A retaining ring is employed which securely positions the wafer under the carrier during polishing. The retaining ring also helps to achieve uniform material removal, especially at the periphery of the wafer, by extending the polishing surface beyond the edge of the wafer [26,27]. The retaining ring and the wafer are held by the wafer carrier which rotates in the same direction as the pad, but at a slightly different rotational velocity [28]. The polishing pad incorporates grooves as well as micro-textures on its surface to aid in slurry transport to and from the pad–wafer interface [29,30,31]. In many applications, a conditioner disc is employed atop the pad surface at a given rotational rate and normal force in order to continuously scratch, and thus, help rejuvenate surface micro-texture. When the wafer engages with the pad surface, the abrasive particles in the slurry, along with the chemicals and pad asperities, provide the chemical and mechanical action necessary for material removal that causes local and global surface planarization. To help improve global surface planarization, the wafer carrier head employs a multi-zone pressure control in order to radially adjust the applied pressure on the back of the wafer [32].

In a CMP process, much like violin playing, as the wafer, the slurry nano-particles and the pad make physical contact with one another, multiple high-frequency stick-slip events are created that cause vibrations within the wafer-slurry-pad-polisher system. These vibrations are manifested in the form of fluctuations of shear and normal forces during polishing. If these forces can be accurately and precisely measured (not many polishers used today in the industry can do this), fast fourier transformation (FFT) may be performed to convert the force data from the time domain to the frequency domain in order to quantify the frequency distribution and the amplitude of the measured forces. Previous studies by our research team have shown the benefit of using such spectral analyses methods. Sampurno et al., reported that interactions between the wafer and abnormally large abrasive particles that were intentionally spiked in the slurry, enhanced the spectral amplitude of the shear force at low frequencies [33]. The same study showed that when the wafer interacted with small abrasive particles, the spectral amplitude tended to shift to higher frequencies. Another study reported that unique and consistent spectral fingerprints were generated showing significant changes in several fundamental peaks during the early evolution of wafer topography and subsequent layer transition to silicon nitride during shallow trench isolation CMP [34]. During barrier CMP, unique and consistent spectral fingerprints were again shown to be generated from shear force data showing significant changes in several fundamental peaks before, during and after TaN clearing [35]. Han et al. employed the same method to monitor the progression of pad break-in and the effect of various pad conditioning schemes in real-time [36,37]. Our present study continues to explore a combination of the unique spectral fingerprinting methods noted above, to simply demonstrate that certain well-established violin characteristics and test methods have counterparts in CMP.

## 2. Parallels in Violin Playing and Wafer Planarization

Below, we summarize some of the parallels that we believe exist between playing the violin and polishing a patterned wafer:

- Bow-string relative velocity ↔ Pad-wafer relative velocity.
- Bow normal force ↔ Wafer normal force.
- Horse hair ↔ Wafer.
- Bow ↔ Wafer carrier head.
- Bow tip, frog and screw ↔ Retaining ring.
- Rosin ↔ Slurry.
- String ↔ Pad.
- Four strings ↔ Four platens.
- Bridge ↔ Sub-pad for each platen.
- Repeated post-performance application of rosin ↔ Ex-situ conditioning.
- Bow drift velocity (in-plane) ↔ Carrier head oscillation.
- Bow skewness angle (in-plane), tilt (off-plane) and inclination (off-plane) ↔ Multi-zone pressure control capability of the carrier head.
- Top and bottom plates, ribs and other key components ↔ Polisher’s body.
- Typical hair-rosin-string coefficient of friction (COF) values ranging from 0.3 to 0.8 ↔ Typical wafer-slurry-pad COF values ranging from 0.3 to 0.8.

The ultimate goal of a master violinist is to project the highest quality sound to the listener well knowing that his or her talents will always be insufficient, as the instrument, hall acoustics, proximity to the audience and many other factors hugely affect the overall listening experience. When it comes to the instrument itself, certain characteristics (that are not necessarily independent of one another) such as impact-induced mobility, bow-induced mobility, sizzle, directivity, projection and the like have been identified and quantified to help objectively compare one violin’s performance to another [8,12,18]. Here, we set out to demonstrate that some of these properties and test methods have strong counterparts in CMP, especially its kinematic and mechanical aspects, which when applied to the process, can help baseline, predict, and even improve planarization performance and improve key wafer-level metrics.

## 3. Mobility Plots

**Figure 1:**
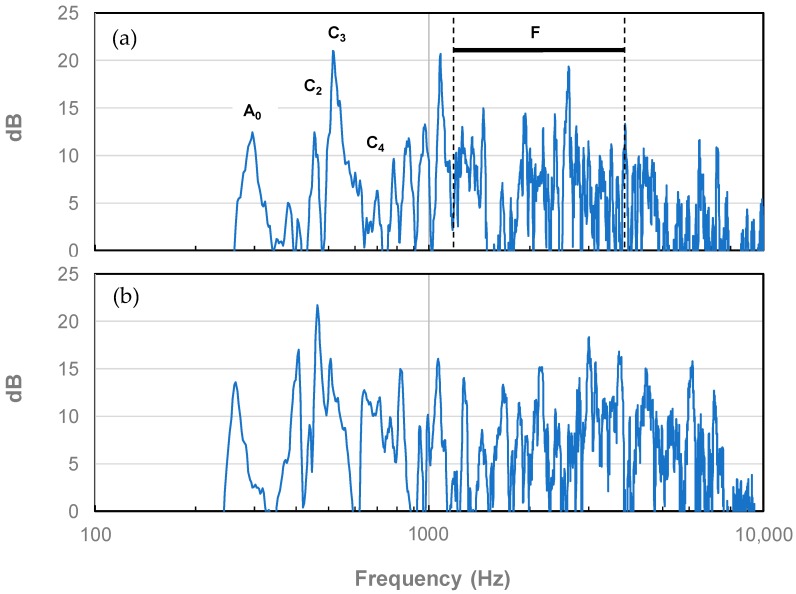
Mobility plots of the “Plowden” del Gesù (1735) (**a**) and a good commercial violin (**b**).

The mobility plot is essentially a transfer function where one induces a vibration, and in the case of the violin, creates a sound. The mobility plot of arguably one of the greatest surviving violins, the “Plowden” Guarneri del Gesù (1735) is shown in Figure 1a as replotted by us using raw data provided by Zygmuntowicz [38] and based on tests performed by Bissinger and Oliver [16]. These types of spectra are obtained through the excitation of the base edge of a violin’s bridge by multiple sinusoidal hammer strikes as described in detail by Dünnwald [8] and others.

The main frequency peaks previously identified as being critical to a violin’s quality and denoted as A0, C2, C3 and C4 [8] along with the family of peaks denoted as “F” by the same researchers are highlighted. Features distinguishing the “Plowden” from other violins may be described as follows:

- The high amplitude and isolated A0 peak at approximately 300 Hz corresponding to air flow in and out of the f-holes.
- The high amplitude and isolated C2 peak at approximately 460 Hz representing strong motion of the top plate.
- The high amplitude and isolated C3 peak at approximately 520 Hz corresponding to strong 2-dimensional motion of the top and bottom plates.
- The suppressed and isolated C4 peak at approximately 690 Hz along with its low-magnitude neighboring peaks up to about 1100 Hz which ensure that the violin does not sound boxy and nasal.
- The initially ascending, and then somewhat descending, collection of F peaks in the 1200–4000 Hz range, where the human ear is most sensitive, giving the violin its brilliance and superior radiation and resulting in equal overtones of all sounds and therefore allowing for a certain “evenness” [8] at the lower playing range. According to Meinel [39], having peaks with small amplitudes above 3000 Hz is critical for ensuring “a harmonious softness, and a fine, pure response”.

Figure 1b also shows the mobility plot for a good commercial violin tested under identical conditions to that of the Guarneri (also provided by Zygmuntowicz). Notable differences between the two may be summarized as follows:

- The commercial violin, although having a comparable output at the sub-600 Hz range, exhibits significant shifts in the A0, C2 and C3 peaks to lower frequencies (by approximately 30 Hz–60 Hz) which diminishes sound quality.
- The C4 peak is neither suppressed nor isolated causing the commercial violin to sound somewhat boxy and nasal.
- The commercial violin is much less brilliant as evidenced by the relatively flat collections of peaks in the 1200 Hz–4000 Hz range.
- Peak amplitudes above 3000 Hz are quite high for the commercial violin which take away its harmonious softness and pure response.

**Figure 2:**
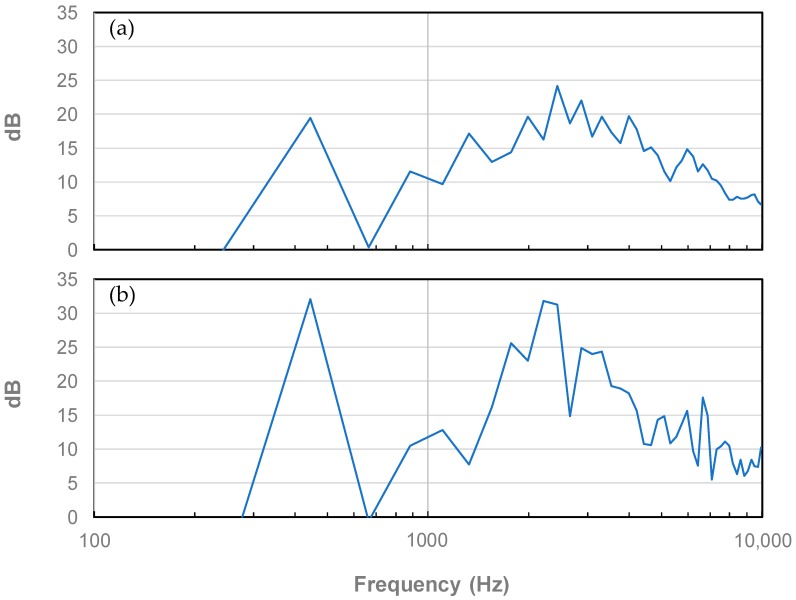
Hammered (**a**) and bowed (**b**) mobility plots of a violin for the A3 note averaged over octave bands.

The published work of Harris [12] takes the mobility plots one step further by comparing impact-induced mobility plots of violins to their bow-played counterparts. The idea here is that played violins will surely have a different (yet somewhat related) spectral fingerprint that should take precedence over hammer-induced mobility due to the simple fact that audiences have always paid to listen to a violin being played rather than its bridge getting struck by a small hammer. Having said that, it is nearly impossible to establish a best-known-method (BKM) for bowing a particular string (e.g., choices involved in the bow and the tension on the hair, string (and its tension), temperature, rosin, normal force, sliding velocity, inclination, tilt, skewness, drift velocity, and the like) and then repeat the method hundreds, if not thousands of times on hundreds of violins to be tested. That is why impact-induced mobility diagrams continue to be the *de facto* standard. Figure 2 compares the hammered mobility curve of a modern violin to its bowed mobility counterpart when the A3 note is played on the G-string. One can see that, at lower frequencies, the impact and bowed mobility peaks are more or less aligned and roughly of the same amplitude. At higher frequencies, where the human ear is most sensitive, peak alignment becomes less and less obvious, and the amplitudes of the bowed mobility peaks rise, giving the peaks more definition, and the violin, a greater “sizzle” [18]. 

Inspired by the periodic hammer strike tests conducted first by Backhaus [1,2] and Dunnwald [8], among others, and knowing that no such tests had been previously reported in the CMP-relevant literature, we decided to take a rather large rubber-felt hammer and strike our APD-800 polisher (Araca, Inc., Tucson, AZ, USA) [40] at the center of the trailing edge side of the wafer carrier head. Our polisher is equipped with force transducers suitable for acquiring real-time shear and normal forces at high frequencies [40]. To measure the shear force, a load cell is installed in the wafer carrier system which itself is constructed above a stainless-steel plate attached on top of the rigid frame of the polisher. Between this plate and the rigid frame of the polisher, there are two parallel sliders that confine plate movement to an axis that is perpendicular to the center of the pad and the center of the wafer. A shear force load cell is then installed to restrict such movement and, at the same time, to measure forces in that particular direction. The shear force generated between the pad and the wafer during polishing is transferred and registered on the load cell. In addition, the body of the polisher is set-up on top of 4 larger load cells that measure instantaneous normal force. During measurement, the load cells convert the actual force into a voltage signal (a linear correlation exists between the two) which is then amplified and recorded. All input and output parameters associated with the polishing process are automated, controlled and monitored via a dedicated computer running on a proprietary software developed specifically for our purposes. The computer also synchronizes the friction table to the polishing process so that the real-time shear and normal force data can be obtained at 1600 Hz acquisition frequency and reported as instantaneous shear force and normal force. Fast fourier transformation (FFT) is then employed to convert the fluctuating force component of the measured total unidirectional shear force (or normal force) from the time domain into the frequency domain [33,34,35]. For our polisher, the maximum frequency that can be deduced is 800 Hz which is the Nyquist frequency of sampling rate at 1600 Hz. The Nyquist frequency is the maximum frequency that can be computed at a given sampling rate in order to be able to fully process the shear force signal without any aliasing problems [41,42]. This method has been described in detail elsewhere [33,34,35].

**Figure 3:**
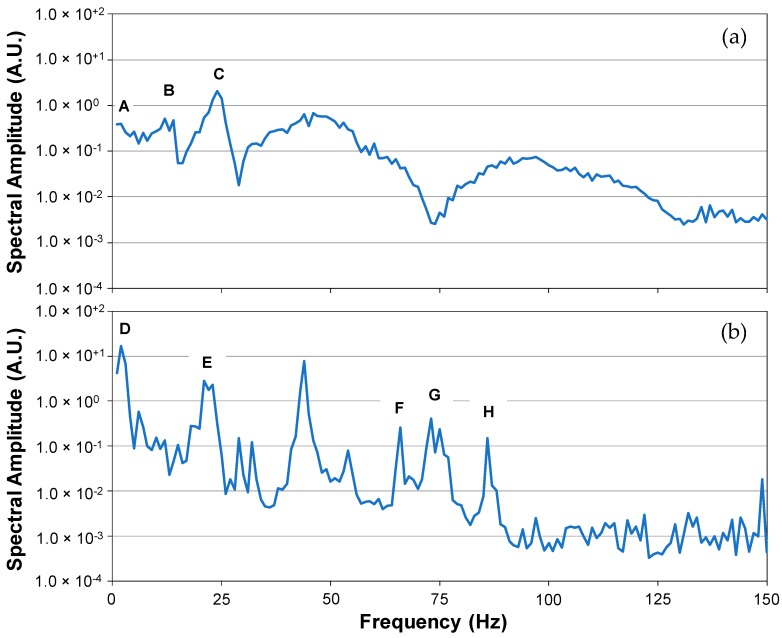
Mobility plots (based on shear force) corresponding to our APD-800 polisher (Araca, Inc., Tucson, AZ, USA) during seven periodic hammer strikes (**a**) and during a typical copper chemical mechanical planarization (CMP) process (**b**).

The mobility plots (based on shear force) of the APD-800 polisher, generated from a total of seven periodic hammer strikes, is shown in Figure 3a. We have chosen to truncate the *x*-axis at 150 Hz because the spectral amplitudes larger than 150 Hz are too low for the actual CMP processes to provide any useful information and can most likely be considered as noise [33,34,35,36,37]. Results show three major peaks, denoted as A–C:

- A: 1 Hz–2 Hz with a spectral amplitude of 4.0 × 10^−1^,
- B: 12 Hz–14 Hz with a spectral amplitude of 5.1 × 10^−1^, and,
- C: 23 Hz with the highest spectral amplitude at 1.41 followed by its widened harmonic peak averages at approximately 46 Hz and 92 Hz.

It is important to note that the baseline spectrum (i.e., one without any hammer strikes) did not exhibit any vibrations at these 3 frequencies. As such, the peaks identified above are independent peaks that can indeed be attributed to the polisher’s natural resonance. In some cases, the peaks are possibly due to the fact that the polisher is more than 2500 pounds in weight and quite complex and asymmetrical in its design with hundreds of ceramic, plastic and metallic components. Also, because of this mechanical complexity and material variety, and the fact that our “hammer-impulse” method was our very first attempt, we do not know which peaks are significant as there are no published comparisons. Figure 3b shows a typical mobility plot obtained during a copper CMP process using a blanket 300-mm copper wafer polished on an Epic^®^ D100 concentrically grooved pad (manufactured by Cabot Microelectronics Corporation, Aurora, IL, USA). The copper wafer was polished for 60 s with PlanerLite 7105 slurry (manufactured by Fujimi, Kiyosu, Japan) mixed to its recommended ratio with hydrogen peroxide. Slurry flow rate was kept constant at 250 mL/min. A 3M Trizact B5 conditioning disc (3M Company, St. Paul, MN, USA) was used to condition the pad at a constant normal force of 27 N. The conditioning disc rotated at 95 rpm with a sweeping frequency of 10 times per minute across the pad surface. The disc, pad and wafer rotated counter clockwise. The wafer polishing pressure and pad-wafer sliding velocity were 2.3 psi and 1.6 m/s, respectively. The pad was initially broken in for 1 h with ultra-pure water. Ten dummy copper wafers were then polished for a total of 10 min to ensure that a stable pad surface had been achieved prior to polishing the copper monitor wafers. Similar to the work published by Harris [12] and shown in Figure 2, we see that polishing a wafer is much like playing a violin with a bow in that at lower frequencies the hammered and polished mobility peaks are more or less aligned. On the other hand, at higher frequencies, peak alignment becomes less and less obvious and the peaks become more isolated and defined in the case of the polished wafer spectrum. Here, the polisher’s resonance takes on a life of its own giving the system a much greater “sizzle”. The polished wafer spectrum shows primary peaks, denoted as D–H:

- D: 1 Hz–2 Hz with a spectral amplitude of 17. Although this peak happens to coincide with the polisher’s natural resonance (at peak A), it is undoubtedly an independent peak caused by the collective motion of the platen, the carrier and the conditioning disc (rotational velocities of the 3 range from 66 rpm to 95 rpm).
- E: 21 Hz–23 Hz with a high spectral amplitude of 3. The frequency is consistent with (and probably because of) peak C in the hammered case having a pronounced harmonic peak at approximately 44 Hz.
- F, G and H: At approximately 66 Hz, 74 Hz and 86 Hz, respectively; peak G has a harmonic peak at approximately 149 Hz. All three fundamental peaks are somehow due to the interactions of the wafer with the pad’s micro-texture and the slurry nano-particles although no clear causes can be attributed to them at this point.

## 4. Directivity

The directional characteristics of sound radiation for a violin is critical from a listener’s point of view. Directivity, which is measured as the violin is being played, is a dimensionless parameter representing the variance of forces exerted on the top plate of a violin divided by the variance of forces on its bottom plate [16]. This parameter has been successfully used by many violin researchers as a measure of directional sound radiation [16,18,43,44,45]. Values close to unity represent isotropic and omnidirectional sound (e.g., suitable for listeners of a Beethoven string trio in close quarters where the soundscape can be intense and detailed), while values higher than one indicate that the violin’s sound can be heard over a hundred or so other instruments being played simultaneously while unidirectionally carrying across long distances to reach listeners seated far away (e.g., suitable for listeners of the Brahms violin concerto). Directivity has been shown to be between 0.95 and 1.15 for just about all violins (**Old** Italian as well as commercial violins) at low frequencies (i.e., less than 400 Hz). Beyond this, directivity for commercial violins increases rapidly and reaches 1.35 at about 900 Hz and then rises very slowly to about 1.45 at approximately 4000 Hz. On the other hand, when it comes to Old Italian violins, past 400 Hz, directivity increases rapidly, reaching values of about 1.65 at approximately 900 Hz and then continues to rise further to about 2.45 at 4000 Hz [16,44].

It is the presence of this asymmetry in force variances on the top and bottom plates (which manifest themselves into uneven 3-dimensional plate vibrations), and the resulting anisotropy in sound radiation at moderate to high frequencies that has inspired us to investigate whether a parallel metric exists in CMP that can be beneficially exploited. From 1927 to the present day, all CMP models for planarization rate and blanket material removal have successfully incorporated the average coefficient of friction (i.e., the average value of shear force divided by normal force) with no consideration whatsoever of the variance (or fluctuations) of such forces. Given the fact that our polisher is capable of successfully measuring forces in both directions (up to 1600 times per second), below we set out to measure this CMP-specific parameter, which here we will refer to as Δ, and qualitatively correlate it with copper blanket film removal rates at selected pressures and velocities.

**Figure 4:**
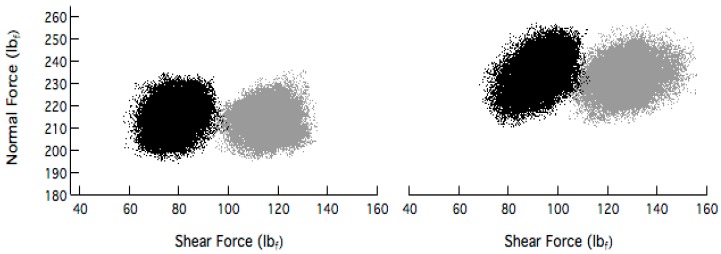
Shear force and normal force scatter plot for copper CMP processes resulting from Disc A (gray) and Disc B (black). The wafer polishing pressures and pad-wafer sliding velocities are 2.1 psi–1.4 m/s (**left**) and 2.3 psi–1.6 m/s (**right**).

Figure 4 shows scatter plots of the instantaneous normal force and shear force measured during several 1-min blanket 300-mm copper wafer polish processes on an Epic^®^ D100 concentrically grooved pad. At two different combinations of pressure and velocity. Wafers were polished with the PlanerLite 7105 slurry mixed to its recommended ratio with hydrogen peroxide. Slurry flow rate was kept constant at 250 mL/min. Half of the wafers were polished with a 3 M Trizact CVD diamond-coated conditioning disc (Disc A). A Morgan Advanced Materials CVD diamond-coated conditioner (Disc B) was used to polish the second half of the monitor wafers. In all cases a constant normal force of 27 N was applied to the disc which rotated at 95 rpm with a sweeping frequency of 10 times per minute across the pad surface during in-situ conditioning. The disc, pad and wafer rotated counter clockwise. For each force cluster shown, there are a total of 60,000 data points from which values for shear force and normal force variances can be easily measured and their ratio calculated to give a value for directivity, Δ.

Table 1, shows the values for Δ and copper removal for the 4 cases tested. Results indicate that for a given pressure and velocity combination, the value of Δ associated with a process using Disc A is significantly higher than that using Disc B. Moreover, the corresponding values for removal rate are also higher, thus indicating the possible presence of a qualitative, albeit loose correlation between the two metrics. To the first order, this correlation makes sense since in CMP, material (generally in the form of a chemically softened surface layer) is removed as a result of 3-body contact events among the wafer, slurry nano-particles and pad asperities, and by the relative sliding action of the wafer, in the direction of shearing. The fluctuations in shear force are due to numerous stick-slip events at high frequencies which combine to strip away the chemically softened surface layer bit by bit.

On the other hand, fluctuations in normal force are caused by tiny vertical displacements in the collective carrier head, wafer, pad and platen assembly. Among other things, these fluctuations may be:

- Hardware related; possibly due to the gimballing action of the carrier head, or slight performance mismatches among myriad hydraulic pistons, gears and bearings,
- Pressure control related; possibly due to the inherent feedback control mechanisms of the numerically controlled systems, or,
- Consumables related; possibly due to density, vertical compliance and rebound differences in various regions of the rotating pad, the irregular shapes of the nano-particles, morphology of the film being polished, and the uneven wetting of the surfaces by the slurry.

Anyhow, such possible variations in normal force, at best, should play a lesser role (if not act as a “loss” function that acts against our objectives) compared to shear-related events. These events would suggest that larger values of Δ, and a greater anisotropy, ought to be preferred. The authors wish to caution that, in our studies regarding this new parameter, that we have coined as Δ, we have only analyzed a very small subset of shear and normal force scatter plots that we have collected over the past 4 or so years. Our next goal (the work has already begun) is to systematically construct shear force and normal force scatter plots from data collected in the past 4 years for copper and tungsten applications to extract values of directivity for each case in an attempt to correlate Δ with pad micro-texture, pad-wafer contact information and removal rate data.

## 5. Conclusions

This study drew upon various acoustic and spectral analysis methods employed by violinmakers and physicists who study Old Italian violins, as well as chemical mechanical planarization (CMP) applications. Driven by a standard violin test whereby a small hammer is used to strike the base edge of a violin’s bridge, we were able to produce mobility plots of our polisher through repeated hammer strikes on the wafer carrier head of our CMP polisher. Fast Fourier Transformation was performed to convert the force data from the time domain to the frequency domain. Results show three independent major peaks (i.e., at 1 Hz–2 Hz, 12 Hz–14 Hz and 23 Hz) which could be attributed to the polisher’s natural resonance. Some peaks were possibly due to the fact that the polisher was very heavy and had a complex and asymmetric design containing hundreds of components. We compared our CMP hammer study to vibrations from an actual wafer polishing process. Similar to hammered and bowed violin tests, at lower frequencies the hammered and polished mobility peaks were more or less aligned. The peak at 1 Hz–2 Hz coincided with the polisher’s natural resonance from the hammer study, however, it was undoubtedly an independent peak with its high spectral amplitude caused by the collective motion of the platen, the carrier and the conditioning disc. The peak at 21 Hz–23 Hz on polished wafer spectrum represented the polisher’s natural resonance as evident in the “hammered” case. At higher frequencies, peak alignment became less obvious and the peaks in the spectrum became more isolated and defined, in the case of the polished wafer. The peaks of the polished wafer spectrum at 66 Hz, 73 Hz and 86 Hz were fundamental peaks and believed to be generated due to interactions among the wafer, pad and abrasive particles in the slurry. We also introduced another parameter from the violin study called directivity, Δ, into CMP. As a key quality metric for the violin, directivity is a dimensionless parameter representing the variance of forces exerted on the top plate of a violin divided by the variance of forces on its bottom plate. The higher a violin’s directivity, the greater its sound isotropy and radiation. Similarly, for CMP, we defined and calculated directivity as the ratio of shear force variance to normal force variance acquired during the polishing process. Our results showed that under the same polishing conditions (i.e., pressure and velocity), the value of directivity increased with copper removal rate. In this study, we demonstrated that the violin’s directivity and mobility have strong counterparts in CMP which when applied to the process, may help baseline, predict, and even improve planarization performance.

## Figures and Tables

**Table 1:** Directivity and removal rate data for selected polishing conditions.

Polishing Pressure (psi)	Pad-Wafer Sliding Velocity (m/s)	Directivity, Δ		Removal Rate (Å/min)	
		Disc A	Disc B	Disc A	Disc B
2.1	1.4	1.48	1.15	5814	4779
2.3	1.6	1.62	1.13	6902	5981
